# Lokomat vs. Conventional Therapy—Impact on Gait Symmetry in Hemiparetic Patients: Preliminary Clinical Study

**DOI:** 10.3390/healthcare13080929

**Published:** 2025-04-18

**Authors:** Marina Potašová, Peter Mačej, Eva Moraučíková, Patrícia Shtin Baňárová, Peter Kutiš

**Affiliations:** 1 Faculty of Health, Catholic University of Ružomberok, 034 01 Ružomberok, Slovakia; marina.potasova@ku.sk (M.P.); moraucikova@ku.sk (E.M.); peter.kutis@ku.sk (P.K.); 2 Rehabilitation Institute in Hrabyně, 747 67 Hrabyně, Czech Republic; 3 Faculty of Humanities, Tomas Bata University in Zlín, 760 01 Zlín, Czech Republic; 4 Faculty of Healthcare, Alexander Dubček University of Trenčín, 911 01 Trenčín, Slovakia; patricia.shtin@tnuni.sk

**Keywords:** hemiparesis, gait reeducation, Lokomat, conventional therapy

## Abstract

**Background/Objectives**: One of the primary goals of neurorehabilitation after stroke is gait reeducation, as it provides the patient with greater autonomy and enhances their safety in daily activities. A preliminary clinical study was undertaken to determine whether robotic gait reeducation using the Lokomat device is more effective than conventional therapy in achieving gait symmetry. **Methods**: The research group consisted of 107 patients, with an average age of 63.54 years, all in the subacute stage of hemiparesis. These patients underwent 4 weeks of neurorehabilitation and were assigned into experimental and control groups. The patients in the experimental group underwent neurorehabilitation (20 sessions) and twice-weekly walking on the Lokomat device (10 sessions). The control group received equivalent neurorehabilitation and conventional gait reeducation. We monitored the return of ideal limb loading (to a 50:50 ratio) and the restoration of the step length on the paretic limb to a physiological length (73 cm), as well as the subsequent restoration of gait symmetry. The measurements were performed using the HP Cosmos Zebris Treadmill FDM-T device. The Wilcoxon Signed Rank test was conducted within each group to analyze the effectiveness of gait reeducation before and after therapy. To compare the results between the two groups, the Mann–Whitney test (α = 0.05) was employed. **Results**: There was no significant difference between the robotic and conventional therapy groups (*p* = 0.432 (>0.05)). A significant change occurred only in the control group in the 50:50 limb loading parameter (*p* = 0.042). There were no significant changes in the other parameters. **Conclusions**: Under the conditions of our study, robot-guided gait reeducation did not appear to be more effective than conventional therapy. The monthly duration of gait reeducation is insufficient to achieve a symmetrical gait in patients with spastic hemiparesis.

## 1. Introduction

Walking is a cyclic locomotor activity consisting of the constant repetition of basic elements, steps [1]. It serves as an indicator of health [2].

Bipedal walking, characteristic of humans, is achieved through the pelvic girdle and lower limbs, accompanied by movements of the trunk and the upper limbs [3]. It is characterized by an orthograde posture, with typical function of the lower limbs and coordinated movements of the trunk, head, and upper limbs. This innate, crossed stereotype ensures constant interaction between the nervous, musculoskeletal, and cardiorespiratory systems [4]. An important factor in bipedal walking is the constant maintenance of balance, which is ensured by the motor cortex and the cerebellum. Since walking is a cyclical activity, its basic unit is the step cycle, a two-step stride [5]. From the perspective of a gait cycle analysis, we can assess both time and distance variables. The time variables are the stride time (the time between successive initial touches of the same foot) and the step time (the time between the initial contact of one foot and the initial contact of the contralateral foot) [6]. The distance variables are the stride length, step length, and step width. Stride length refers to the distance between successive initial contact of the same foot, and the average stride length is 1.44 m. Step length refers to the distance between the initial contact of one foot and the initial contact of the contralateral foot with the ground, and the average stride length is approximately 70 cm. Step width refers to the mediolateral space between the two feet, specifically the distance between the right and left heels when walking. The average normal step width ranges from 8 to 10 cm [6]. The other variables characterizing gait include cadence (the number of steps taken per minute) and gait speed (determined by cadence and stride length, measured in units of distance per time (m/s)) [7]. Another important parameter of a bipedal gait is the load on the limbs. During the gait cycle, both lower limbs should be symmetrically loaded at a 50:50 ratio [8]. The parameters that characterize the step cycle are symmetrical in a healthy individual. But changes in these can occur with any handicap [9]. In particular, their asymmetry is a symptom of many diseases and is particularly prominent in stroke [10,11,12].

Stroke is the acute onset of a central neurological lesion of vascular origin (ischemic or hemorrhagic) [13]. It is a very common and serious disease [14]. Stroke can result in a variety of changes: cognitive, motor (weakness, the loss of voluntary movement), sensory, and proprioceptive (which can affect balance) [15]. Additionally, stroke is a leading cause of serious long-term disability [16]. A major consequence of stroke is gait dysfunction, which often leads to significant disability. Gait impairment is present in the majority of patients who have experienced a stroke [17]. Li reported that gait dysfunction occurs in more than 80% of stroke patients [12].

One’s walking ability following a stroke is characterized by a significant clinical manifestation of asymmetry when compared to healthy people. This asymmetry is clinically manifested by shortening of the stance phase and lengthening of the swing phase on the paretic side [18]. Furthermore, stride length is shortened and walking speed is reduced [17]. Solanki et al. (2018) reported that stroke reduces the weight-bearing capacity of the lower extremities by up to 43% [19]. As a result, gait disorders can cause difficulties in performing activities of daily living [12]. Motor rehabilitation is essential for helping patients with hemiparesis regain their motor function, including their gait [17].

Rehabilitation treatment of a stroke patient begins as soon as possible and focuses on addressing motor and muscle tone disorders, sensory disorders, and the manifestations of these disorders in daily activities (ADLs). Gait reeducation is one of the most important tasks of rehabilitation and has long been a frequent subject of various research studies [20]. The optimal stroke rehabilitation combines targeted training of the motor and sensory functions. Multisensory stimulation of the proprioceptors and exteroceptors replaces the missing impulses from the central nervous system and activates the relevant interneurons, which in turn activate the peripheral motor neurons [21]. Flašková recommends starting rehabilitation on the second day after an incident or as soon as a patient’s basic vital functions have stabilized [21]. Regaining lower limb function is extremely important to gait, as their structural insufficiency and functional impairment can predispose patients to injuries [22].

Gait reeducation is an integral component of comprehensive stroke rehabilitation, and it begins in the acute phase of stroke. During this phase, the physiotherapist selects specific kinesiotherapy techniques for the lower limbs to restore walking function. Targeted locomotor training of the lower limbs is reported as an important factor in the restoration of walking function. It requires the coordination of several motor and cognitive functions, e.g., maintaining balance (postural correction), supporting the transfer of body mass to the paretic limb during the standing phase, reeducation of the swing phase on the stepping limb, and the use of a forward driving force. The cognitive prerequisites for gait training include motivation, the ability to apply control strategies, and the capacity to learn and evoke motor patterns [20]. Several rehabilitation techniques have been proven effective in gait rehabilitation in stroke patients. In recent years, the effectiveness of various mechanical and electronic systems for supporting the gait of hemiparetic patients has been highlighted. Despite this fact, in the Czech Republic and Slovakia, but also in other European countries and worldwide, conventional gait reeducation procedures are still applied. Our main question was therefore whether patients who do not have the opportunity to undergo robotically guided gait reeducation receive equivalent therapy to those patients who do. Which type of reeducation is more effective for a patient’s “first targeted steps”—for restoring the standing and stepping phases of the gait cycle? Currently, despite the existence of several analyses of Lokomat therapy and conventional therapy, at the moment, there is insufficient evidence to conclusively advocate in favor of or against the use of the Lokomat [23].

In conventional gait therapy, the main methods used in neurorehabilitation are the Kabath, Bobath, and Perfetti methods [23]. Specifically, activities like trunk control exercises, load balancing, exercises for static and dynamic balance, and walking training applied through these methods have positive effects on gait reeducation in hemiparetic patients [24,25].

On the other side, the Lokomat is a device that provides exoskeletal robotic gait training [26]. It combines a treadmill with body weight support (a BWS system) while providing exoskeletal support to the patient [27,28]. The exoskeleton supports leg movements during the gait cycle according to a longitudinal and predefined pattern [28]. The Lokomat meets the demanding criteria for contemporary neurorehabilitation, which are based on our understanding of the central nervous system’s plasticity. This device assists the patient in reproducing the various phases of the physiological gait cycle, mobilizing their joints, symmetrically balancing the load, and transmitting proprioceptive afferents. Studies have shown that the rhythmic and repetitive step pattern provided by robotic guidance, combined with active limb loading and kinematic coherence, supports the plasticity of the gait pattern generator, facilitating motor schemes and promoting neural plasticity at both the spinal and supraspinal levels [23]. This method of gait reeducation has quickly gained popularity, as the manufacturers indicate its strong and promising therapeutic modality for improving gait function in hemiparetic patients [26,29], which also facilitates the work of physiotherapists. Several studies have described the mechanisms of the influence on gait in the Lokomat and its advantages over those of conventional therapy and confirmed significant results for the Lokomat in terms of the symmetrical stepping patterns when using the Lokomat with provision of the BWS [30,31,32] and increases in speed [33,34]. On the contrary, some studies published in recent years have stated that the Lokomat is not a superior therapy, especially when it does not have appropriately set walking parameters [23,33]. Baronchelli et al. also stated that it is not definitely clear whether the Lokomat leads to better outcomes compared with those of other more conventional gait rehabilitation methods [23].

Based on these findings, the present study compares the effectiveness of robotic and conventional gait reeducation in patients in the subacute stage of stroke.

The primary aim of this study was to determine whether there was a difference in the improvement in step length and the loading of the paretic limb in patients who underwent Lokomat therapy compared to conventional gait retraining. The secondary objectives were to compare the changes in the load and step length between the two groups; to determine whether the use of the Lokomat is a more effective therapy than conventional gait reeducation in improving walking in patients with spastic hemiparesis; and last, but not least, to obtain results that could help doctors and physiotherapists in deciding which method of therapy is more suitable for individual patients.

## 2. Materials and Methods

A preliminary clinical study of two parallel groups, Lokomat and conventional gait reeducation groups, was undertaken. This study was conducted in accordance with the principles of the Declaration of Helsinki and approved by the Ethics Committee of the Central Military Hospital, No. ÚVN-40-39-2024.

### 2.1. The Study Design

The patients were randomly assigned into two groups: Lokomat and conventional gait reeducation. Our basic inclusion criteria were as follows: patients in the subacute stage of stroke with hemiparesis who were able to walk alone/or with assistance for at least 10 m. The interventions involved the same frequency and duration of therapy for both groups, but for the experimental group, robotically guided gait reeducation on the Lokomat device was undertaken. For the control group, the conventional physiotherapy focused on improving walking. The outcome measures were the deficit in the step length from the physiological value (73 cm) and the deficit in the load from the physiological level (50:50), as measured using the HP Cosmos Zebris Treadmill FDM-T device. The data analysis involved statical testing (the Wilcoxon Signed Rank test and the Mann–Whitney test) to compare outcomes. The effect size was used to describe the magnitude of the difference between the two groups or the strength of an association between variables (ά = 0.05).

A detailed description of this study is provided in the following sections.

### 2.2. The Study Population

**Figure 1:**
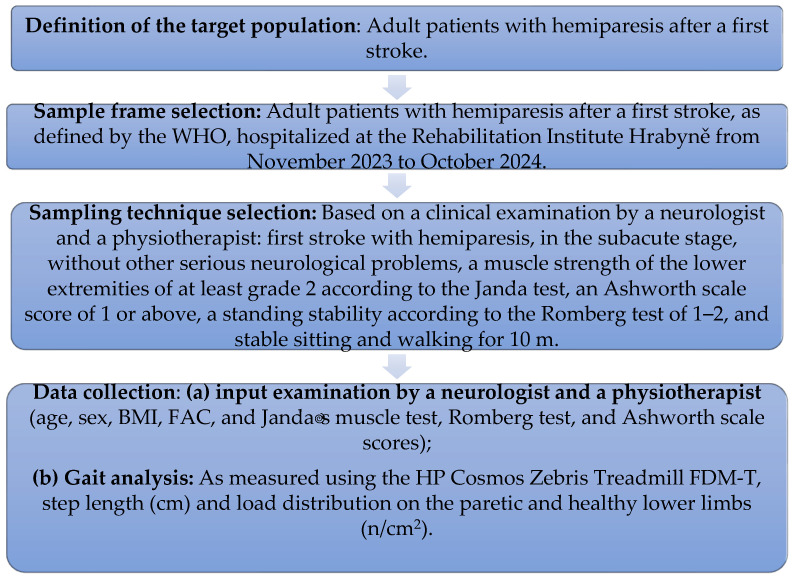
Study sample selection.

Potential participants were suggested by doctors—neurology specialists—in cooperation with a physiotherapist working at the Rehabilitation Institute Hrabyně in the Czech Republic, where this study was conducted. Potential participants were selected based on an initial examination. The main inclusion criteria were being in the subacute phase of a first stroke and exhibiting clinical symptoms of hemiparesis, as defined by the World Health Organization. Patients had to meet the following inclusion criteria as well: (a) being adult patients aged 18 and over; (b) having no history of a previous stroke or neurological or orthopedic disorders; (c) being able to walk independently prior to the stroke and with no serious medical conditions; (d) being cognitively oriented and able to cooperate; (e) experiencing hemiparesis with a lower limb strength level of 2 or more according to the Janda test; (f) being within an interval between the stroke and the initiation of rehabilitation of at least 28 days but no longer than 200 days; and (g) having the ability to sit steadily and walk alone or with assistance for 10 m. The exclusion criteria were as follows: (a) a muscle strength of less than grade 2 in the Janda muscle test; (b) plegia of the affected lower limb or a muscle tone higher than 2 on the Ashworth scale; (c) the inability or unwillingness to cooperate; and (d) having the susceptibility to collapse during verticalization with the risk of falling. The sample size was calculated. It was determined that the minimum number of subjects that needed to participate in this study to have a representative sample of the studied population was 105 people or more. The sample selection process was performed following the steps depicted in Figure 1.

**Figure 2:**
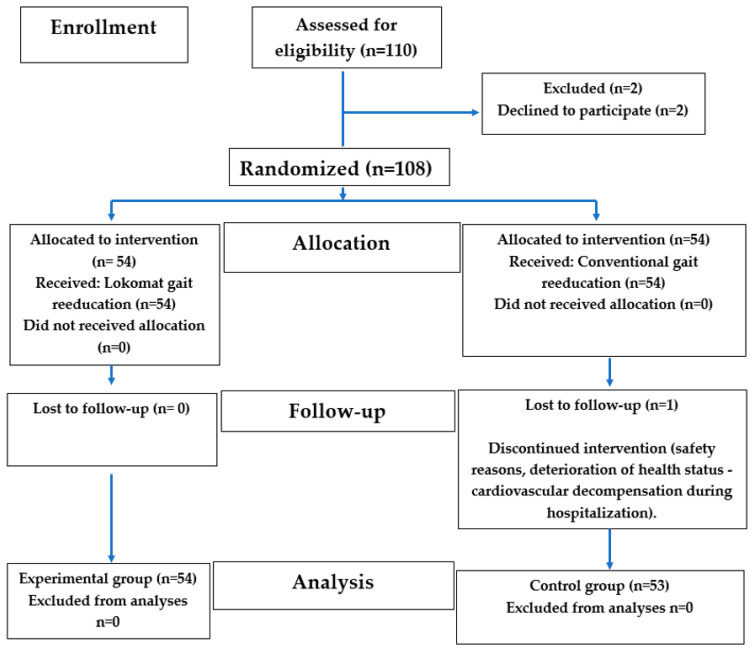
Consort flow diagram.

After the initial examination, if the patients met the above criteria, they were suggested to participate in this study. Potential patients were informed by the doctor and the physiotherapist about the method of group assignment and the course of the treatment in the groups. An open-label (nonblinded) study was conducted. Both the health providers and the patients were aware of the treatment being given. Since patients were randomly assigned into the groups, 2 patients refused to participate in this study. The participants were stratified, as applied in the pilot study by Husemann in 2007 [20], according to diagnosis and lesion hemisphere to minimize an uneven distribution of relevant variables. Different block lengths were used for randomization to ensure the unpredictability of the treatment assignment. Randomly generated numbers were placed into sealed opaque envelopes, based on the selection of which patients were assigned into either the experimental or control group. Each patient was thoroughly informed about this study’s procedures, potential risks, and the option to withdraw from this study. The patients consented to participating in this study by signing informed consent forms. During the study period, 1 patient’s health deteriorated, and this patient did not complete this study. He was excluded from the study sample (see the consort flow diagram in Figure 2).

Figure 2 shows that out of the original 110 participants assessed, 2 declined to participate and were therefore excluded. Subsequently, 108 participants were randomized, of whom 54 were assigned into the intervention group and 54 into the control group. During follow-up, 1 participant from the control group did not complete this study due to deterioration of his health status (cardiovascular decompensation). Ultimately, 107 participants (67 women and 40 men), with an average age of 63.54 years, were analyzed. A total of 54 patients were then in the experimental group, and 53 patients were in the control group. Each patient was properly informed about the course of this research, the risks of this research, and the possibility of withdrawing from this research. The patients agreed to this research. They expressed their consent by signing informed consent forms. The experimental and control groups at baseline are presented in Table 1.

### 2.3. Comprehensive Neurorehabilitation

All of the patients included in this study underwent the standardized neurorehabilitation recommended by the Ministry of Health of the Czech Republic. Neurorehabilitation lasted 4 weeks, which corresponded to the period of treatment for post-stroke patients covered by health insurance in the Czech Republic. Neurorehabilitation was applied every working day for 30 min (Monday to Friday), i.e., 5 days a week. It consisted of Bobath therapy, PNF therapy, and exercises based on neurophysiology. In addition to kinesiotherapy, patients whose conditions necessitated further intervention were also prescribed electrostimulation of the paretic muscles or analgesic currents for painful segments; hydrokinesiotherapy, in the form of exercises in the pool with a therapist for 15–20 min; or the use of Joint Active System (JAS) positioning splints to improve their passive range of motion in the affected joints. Furthermore, the patients underwent speech therapy, psychological treatment, social therapy, inhalation therapy, respiratory therapy, craniosacral therapy (addressing the influence of cerebrospinal fluid), and music therapy. A total of 20 interventions were applied as part of the complex neurorehabilitation program during their hospitalization. The experimental group completed 28.44 ± 1.8 h of comprehensive rehabilitation. The control group completed 28.72 ± 1.4 h (Table 1). Different treatment steps between the groups were implemented in gait reeducation and are presented in the following section.

#### 2.3.1. Treatment Groups

The patients in the experimental group underwent robotic gait reeducation twice a week using the Lokomat device (Lokomat; Hocoma, Volketswil, Switzerland). The basic version of the Lokomat system consisted of the Lokomat (a robotic gait orthosis) and the Lokobase (the body weight support system). It was used in combination with the Zebris treadmill (zebris Medical GmbH; Isny im Allgäu, Germany). The gait training parameters in our experimental group were as follows: Training frequency using the Lokomat: Twice a week. Walking time: Initially 10–30 min and later 20–45 min (average time spent on the Lokomat: 332.67 ± 12.3 min; see Table 1). Walking speed: 1.0–1.5 km/h and later up to 2.5 km/h. Patients’ body weight support: Initially, each patient had their support set at 50% of their body weight. With gradual gait improvements, they attempted to walk without assistance. The load was increased by 5% to 10% for each patient after 5 days of treatment. Biofeedback: Focused on the swing phase, weight distribution, and stride length. Training duration: Four weeks. The patient’s lower limbs were guided according to a pre-programmed physiological gait pattern. Their knee and hip joints were monitored using position and force sensors, allowing for individual adjustments. The torque of their knee and hip actuators was adjusted from 100% to 0% for one or both lower limbs [20,35].

#### 2.3.2. The Control Group

The patients in the control group, in addition to neurorehabilitation, received conventional gait reeducation with a physiotherapist. The patients practiced regulating their trunk stability, their symmetrical loading of the paretic lower limb, the swing of the paretic lower limb, and the symmetry of their step. They attempted to walk down a hallway with a physiotherapist and walking aids, depending on the patient’s tolerance, for 10 to 30 min. In total, the patients completed 317 ± 14 min. of conventional walking during their 4-week hospitalization.

### 2.4. Measurements

An initial gait analysis was conducted before the first rehabilitation intervention, and final measurements were taken during the last session with the patients. The following parameters were assessed before and after treatment: step length (cm) and the load distribution on the paretic and healthy lower limbs (n/cm^2^). Gait was assessed using the HP Cosmos Zebris Treadmill FDM-T (zebris Medical GmbH; Isny im Allgäu, Germany), a system specifically designed for analyzing dynamic gait patterns and integrated with an existing treadmill. It captures and analyzes natural gait movement under a wide range of different conditions. The measurement principles are as follows: The system contains a measuring matrix consisting of capacitive force sensors that are arranged in columns and in lines running closely next to each other. To determine the force distribution across the measuring matrix, the capacity proportional to the force exerted is calculated for each individual sensor. This is achieved through the drive logic, generating a series of sinusoidal burst signals corresponding to the number of columns, via the column decoder, which are then transmitted to the respective measuring columns. The analog signal coupled into the shift register over the lines is proportional to the pressure-dependent capacity. This signal is then forwarded for further processing to the control and signal-processing electronics, from which it is transmitted to a PC and displayed on screen [36].

On the HP Cosmos Zebris Treadmill FDM-T device, the step length of the right and left lower limbs was evaluated. Their sum represents the length of a two-step pattern. The physiological average length of a two-step pattern is automatically generated by the HP Cosmos Zebris Treadmill FDM-T device at 146 cm, with the step length measured at 73 cm. The deficit in the step length (cm) in comparison to the normal physical values was evaluated.

**Figure 3:**
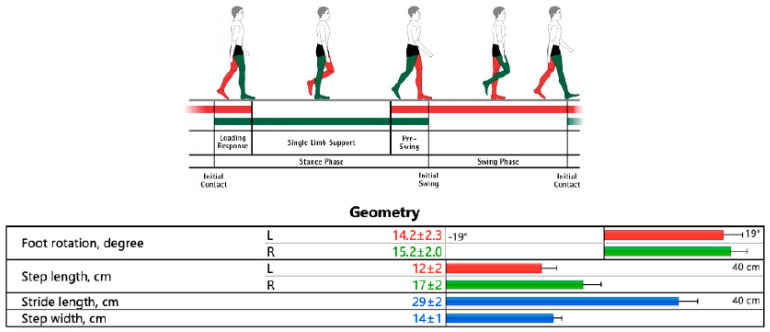
Gait analysis and the results on the HP Cosmos Zebris Treadmill FDM-T (authors’ archive from Zebris Medical GmbH, Isny im Allgäu, Germany).

**Figure 4:**
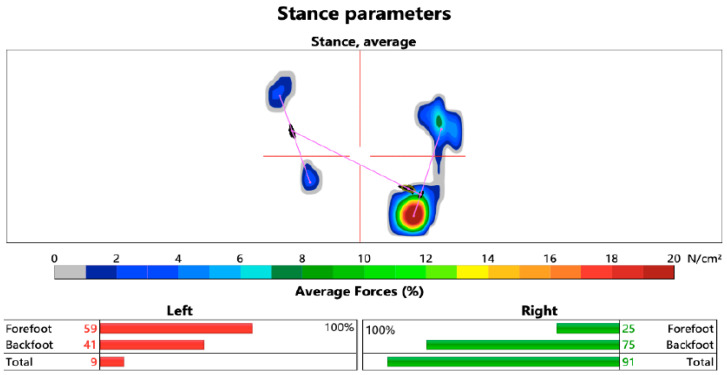
Load distribution during the stance phase of the gait cycle (left hemiparesis) on the HP Cosmos Zebris Treadmill FDM-T (Authors’ archive from Zebris Medical GmbH, Isny im Allgäu, Germany).

As a second parameter, the load on the paretic limb during walking was analyzed. The ideal distribution of weight between the limbs during walking follows a 50:50 ratio. This means that the patient must evenly distribute their weight between the healthy and paretic lower limbs during the stance phase of the gait cycle. Using the HP Cosmos Zebris Treadmill FDM-T device, we evaluated the load on the forefoot and backfoot and the average load on the limbs (average forces) during walking (N/cm^2^) (Figure 3 and Figure 4).

### 2.5. Statistical Analysis

The research results were statistically processed and evaluated in the IBM SPSS program 26. A normality test was performed for the monitored parameters using the Shapiro–Wilk test. Since the data did not follow a normal distribution, non-parametric tests were applied. The variables were tested before and after therapy. The Wilcoxon test was used to assess the equality of the medians pre- and post-therapy in each group. When comparing the results obtained at the end of therapy in the experimental and control groups, the Mann–Whitney test was used to assess the equality of the medians between the two groups, as they represented independent patient groups. The significance level was set at ά = 0.05. The effect size was calculated as well. The effect size was calculated as the mean difference between the two groups + the 95% confidence intervals.

## 3. Results

In the following part of the text, we present an analysis of the changes in step length and the changes in limb loading. Detailed descriptive statistics are included in the Supplementary Materials (Table S1: Data analyses in groups).

### 3.1. The Stride Length Analysis

Table 2 presents the results of the step length measurements before and after gait reeducation in the experimental and control groups and compares these two groups.

Table 2 shows the deviations from the ideal step length before and after gait reeducation for the experimental and control groups. The results of the input and output values, analyzed using the Wilcoxon Signed Rank test, indicate that the difference in the step length from the ideal step length did not significantly change as a result of completing gait reeducation through either the Lokomat therapy or the conventional therapy. The change was not statistically significant in either the experimental group (*p* = 0.191) or the control group (*p* = 0.492). Finally, we compared the monitored gait parameter between both groups. The resulting median deviations were compared. The results indicate that the differences in these two types of gait reeducation were not significant either (*p* = 0.432). A small effect size was also calculated in the groups. As the null hypothesis H_0_ was confirmed and we found a small effect size, we can state that there were no observable differences between the sets.

### 3.2. The Limb Load Analysis

Table 3 presents an analysis of the weight distribution before and after gait reeducation in each group and a comparison of the experimental and control groups.

The results of the input and output values, analyzed using the Wilcoxon Signed Rank test, indicate that after completing gait reeducation, the load on the paretic limb towards the ideal load of 50:50 was not statistically significant in the experimental group (*p* = 0.057), but it was very close to a significant value. However, in the control group, the change in the weight distribution and therefore the load on the paretic limb after conventional gait therapy was statistically significant (*p* = 0.042). Finally, we compared the monitored gait parameter between both groups. The resulting median deviations were compared. The results indicate that the differences in these two types of gait reeducation are not statistically significant (*p* = 0.432). A small effect size was confirmed in the sets.

## 4. Discussion

One of the primary goals of stroke rehabilitation is to regain motor skills [37]. Improving gait is particularly important, as it enhances a patient’s autonomy and increases their safety in daily activities [10].

This study examines whether robotically controlling a patient’s gait using the Lokomat device is more effective than conventional therapy. Although the Lokomat is becoming more and more affordable, there are still clinics that cannot afford it. For example, in Slovakia, 17,000 residents are affected by strokes annually [38], but currently there, are only six clinics that offer robotic rehabilitation. The remaining clinics still only use conventional walking therapy. Therefore, the aim of the study was to compare the effectiveness of these two therapies intended for gait reeducation in patients with hemiparesis. This study also offers an answer to the question of whether patients with hemiparesis who, despite the existing robotic rehabilitation, undergo conventional gait therapy are discriminated against in terms of receiving the most effective therapy.

The comparison of the effectiveness of the Lokomat gait training and conventional neurorehabilitation analyzed in this study showed that there was no significant difference in motor function between the groups before or after treatment (*p* = 0.432). This suggests that under the conditions of our study, robot-guided gait reeducation is not more effective than conventional therapy in terms of regaining walking function. Similar to our findings, several other studies comparing the effectiveness of the Lokomat and conventional therapy have not shown any significant differences in their effectiveness [24,39,40,41]. Dellan even stated that most of the evidence suggests that Lokomat therapy is only as effective as the other standard therapeutic approaches but not better [42]. This has also been confirmed by Bonnyaud, who stated that many studies have been conducted on gait reeducation in patients with spastic hemiparesis following different techniques, but none of these have been shown to be the most effective or preferred [39]. In recent decades, many studies have also been conducted comparing the effectiveness of the Lokomat with that of either conventional therapy, overground walking, or treadmill walking in patients with spastic hemiparesis. Most of the evidence has been collected and analyzed in systematic reviews, which have found that Lokomat therapy is effective but generally not superior to other forms of therapy, such as overground walking or treadmill therapy [42,43,44]. However, on searching these studies, some showed an advantage of the Lokomat over the traditional training methods [45,46], but others failed to and urged therapists to remain cautious about this option [24,47,48].

In this study, we analyzed gait reeducation in patients with spastic hemiparesis during the subacute stage of the disease in terms of the restoration of two gait variables: limb loading and step length. Individuals with hemiparesis due to stroke commonly demonstrate difficulty bearing weight on their paretic lower limb and transferring weight from one leg to the other [49,50]. Reduced weight-bearing on the paretic limb has been associated with functional deficits when rising from a chair [51], standing [52], and walking [53]. The ability to transfer one’s body weight between the lower limbs is related to impaired standing and stepping balance [54,55] and gait performance [49]. Diminished weight transfer onto the paretic limb contributes to gait asymmetry, which often results in increased energy expenditure [15]. Therefore, load training of the paretic lower limb is a basic prerequisite for walking, and every reeducation therapy for walking should begin with load training for the affected limb. The next step in walking reeducation is restoration of the step length. By restoring these two dynamic walking parameters, we establish the prerequisites for reeducating the other elements of walking, ensuring not only symmetrical but also economical walking in a hemiparetic patient. The economization of movement, on the contrary, refers to its symmetrization [3]. Therefore, in this study, we examined whether the forms of neurorehabilitation we chose would affect the load on the paretic and healthy lower limbs to the physiological values at a ratio of 50:50 and whether the step length of the paretic limb would be adjusted to the physiological value (an average physiological length of 73 cm), which is a prerequisite for the acquisition of gait symmetry.

The results of our study show that in the monitored parameters, a significant adjustment occurred only in the load on the paretic limb at a ratio of 50:50 to that on the healthy lower limb in the group of patients undergoing the conventional therapy (*p* = 0.042). Even though the changes in the load distribution after completing the Lokomat gait training were close to a significant value (*p* = 0.057), no significant changes were found in this parameter, nor in the other variables monitored, in either group. This suggests that under conditions such as those in our study, neither robotic gait training nor the standard conventional gait therapy lasting 4 weeks is significantly effective in restoring gait function. The results of our study may have been influenced by several factors: the duration of neurorehabilitation, which lasted only one month; the number of gait interventions completed (Lokomat 10×); the Lokomat’s parameter settings; and the severity of the patients’ motor impairments. Several studies have stated that it takes a long time (through long-term rehabilitation), longer than 1 month, to adjust one’s walking function after a stroke. Li argued that stroke recovery is a journey and, for example, a person with hemiparesis continues to improve in their walking from walking with parallel bars to walking with a cane and then walking without assistance at a moderate speed over the course of 2 years post-stroke [56]. The Mayo Clinic in Arizona, USA, has stated that some stroke survivors recover quickly. But most need some form of long-term stroke rehabilitation. This can last for possibly months or years after a stroke [57]. Another factor that may have caused the non-significant study results in the experimental group was the frequency and duration of the gait training on the Lokomat. In our experimental group, each patient completed 10 sessions of gait reeducation using the Lokomat, 2× per week (an average time on the Lokomat = 5.54 h). But researchers have found that intensive therapy, added to the standard rehabilitation, produces the greatest improvement. When it was given in the subacute period, stroke survivors experienced clinically meaningful improvements after 20 h of additional therapy [58], which is a significantly longer therapy duration than that which our patients completed. Unfortunately, the time spent on the Lokomat was limited by the reimbursement of healthcare by public health insurance. The adjustment of the Lokomat’s settings may also have caused a non-significant improvement in their stride cycles. Several studies have discussed how to set the Lokomat for the most ideal gait reeducation. However, van Kammen claimed that when using a clinically relevant range of settings for the training parameters in Lokomat therapy, only speed had a significant effect on the muscular output in both the affected and unaffected legs. The level of temporal symmetry was not affected by altering the training parameters [27]. In general, it can be said that the ideal features of gait training include practice at treadmill speeds consistently faster than a subject can walk over the ground [59].

Last but not least, the results of this study may also have been related to the patients’ overall health. Dellen and Morone report that there is evidence that more severely affected stroke patients may improve more than less affected patients [42,60]. In our study, patients with a smaller motor deficit were monitored (a muscle strength score according to the Janda test of 2 or more; a spasticity score of 2 or less according to the Ashworth scale); therefore, the changes in limb loading and step length achieved may not have been as significant as expected.

However, based on the findings from this study, we can conclude that hemiparesis is a complex and long-term condition, requiring subsequent long-term therapeutic interventions. The results of this study showed that there were no significant differences between the effectiveness of gait reeducation on the Lokomat device and the conventional therapy. They indicate that patients who are referred for conventional gait therapy do not receive inferior therapy. On the contrary, in the early stages of gait reeducation, especially in terms of restoring the load on the paretic limb, conventional therapy has been shown to be more effective compared to Lokomat therapy.

### This Study’s Limitations

One limitation of this study was the duration of the patient follow-up. This was determined by the length of hospitalization covered by public health insurance in the Czech Republic, and it was not possible to extend this since the patients would have to have paid to extend the therapy as self-payers, which they did not agree to. In addition to the above-mentioned study confirming the necessity of long-term rehabilitation (of over one month), a study conducted by Kelley, in which they monitored 3 months of Lokomat treatment, also stated that there was no significant effect on the monitored gait parameters of the 3-month robot gait treatment [61].

## 5. Conclusions

The results of this study indicate that under conditions similar to ours, robot-guided gait reeducation using the Lokomat device is not more effective than conventional therapy. A significant change occurred only in the control group in the parameter of limb loading at a ratio of 50:50 with that on the healthy leg. No significant changes were observed in the other parameters monitored, neither in the experimental nor in the control group.

Despite the fact that the study results indicate that there were no significant differences between the effectiveness of gait reeducation on the Lokomat device or the conventional therapy, they are significant on two levels: for therapists who work with the Lokomat, as well as for those who do not have a Lokomat in their workplace. For therapists working with the Lokomat, these results may help in deciding which therapy method is most suitable for individual patients. These results also form the basis for modifying treatment with the Lokomat (an initial load on the patient lower than 50%, the daily application of walking on the Lokomat, or its combination with gait training in the conventional manner).

For therapists who do not have the opportunity to work with the Lokomat, the results indicate that conventional gait reeducation is comparable to gait reeducation on the Lokomat. Especially at the very beginning of gait reeducation, targeting gait therapy directly to the motor deficit (e.g., forcing the load on the limb, training step length) may have a more significant treatment result than globally influencing the deficit.

Last but not least, as this was a preliminary study, the results of this study indicate the need for future research to optimize the gait rehabilitation protocols for patients with spastic hemiparesis.

## Figures and Tables

**Table 1:** Experimental and control groups at baseline.

Experimental Group			
	Mean ± SD	n	%
Age	64.31	-	-
Sex	-	M = 12; F = 42	M = 22.22; F = 77.78
Hemiparesis dx.	-	32	59.3
Hemiparesis sin.	-	22	40.7
BMI	27.85 ± 3.8		
Time since stroke (in days)	36.3 ± 7.3		
Romberg test score	2.06 ± 0.2		
Muscle strength DK according to the Janda test	3.19 ± 0.6		
FAC	3.04 ± 0.6		
Ashworth scale score	0.61 ± 0.8		
Time spent on complex neurorehabilitation (in hours)	28.44 ± 1.8 1536 total		
Time on the Lokomat during hospitalization (in minutes)	332.67 ± 12.3 17,964 total		
Control group			
Age	61.23	-	-
Sex	-	M = 28; F = 25	M = 52.83; F = 47.17
Hemiparesis dx.	-	29	54.72
Hemiparesis sin.	-	24	45.28
BMI	29.53 ± 3.6		
Time since stroke	38.3 ± 9.8		
Romberg test score	3.43 ± 0.7		
Janda test score DK	3.34 ± 0.5		
FAC	3.06 ± 0.6		
Ashworth scale score	0.53 ± 0.7		
Time spent on complex neurorehabilitation (in hours)	28.72 ± 1.4 1522 total		
Time spent on conventional gait training during hospitalization (in minutes)	317 ± 14 16,801 total		

**Table 2:** Step analysis.

	Parameter: Step	Mean	Median	SD	*p*	Effect Size
Experimental group	Stride length before rehabilitation	73.3	78.0	29.4	-	
	Stride length after rehabilitation	79.0	74.0	26.8	-	
	Deviation from ideal length before	24.7	27.5	15.5	-	
	Deviation from ideal length after	22.1	20.5	15.8	-	
	Difference in deviation	2.6	3.0	16.2	0.191 •	0.133
Control group	Stride length before rehabilitation	67.5	66	24.0	-	
	Stride length after rehabilitation	76.1	75	27.6	-	
	Deviation from ideal length before	20.1	19.5	14.2	-	
	Deviation from ideal length after	21.3	15.5	17.6	-	
	Difference in deviation	−1.2	1.0	16.2	0.492 •	0.003
Comparison	Deviations in lengths between groups	-	-	-	0.432 ▫	0.234

• Wilcoxon Signed Rank test; ▫ Mann–Whitney test.

**Table 3:** Results of the weight distribution towards the ideal load.

	Parameter: Load	Mean	Median	SD	*p*	Effect Size
Experimental group	Deviation from ideal load before	6.5	5.1	5.6	-	
	Deviation from ideal load after	5.4	4.1	4.6	-	
	Difference in deviation	1.0	0	0.1	0.057 •	0.241
Control group	Deviation from ideal load before	8.7	5.1	10.9	-	
	Deviation from ideal load after	6.3	5.1	6.7	-	
	Difference in deviation	2.3	1	8.1	0.042 •	0.237
Comparison	Load deviations between groups	-	-	-	0.432 ▫	0.015

• Wilcoxon Signed Rank test; ▫ Mann–Whitney test.

## Data Availability

The data are available upon request from the corresponding author.

## Supplementary Materials

The following supporting information can be downloaded at https://www.mdpi.com/article/10.3390/healthcare13080929/s1, Table S1: Data analyses in groups.
