# Supplementary material for: Lokomat vs. Conventional Therapy—Impact on Gait Symmetry in Hemiparetic Patients: Preliminary Clinical Study
Source: Healthcare (Basel). 2025 Apr 18;13(8):929. doi: 10.3390/healthcare13080929 (PMC12027008; doi:10.3390/healthcare13080929)
Supplement: Supplementary file 1 [file healthcare-13-00929-s001.zip › healthcare-3475226-supplementary.pdf]

Supplementary material Table S1: Data analyses in groups

Analysis of deviations from ideal limb loading 50:50					
	statistical indicators	deviation before therapy	deviation after therapy	difference	difference in %
Exper. group	Mean	6.5	5.4	1.0	-25.9
	Median	5	4	0	0
	Mode	3	2	-1	0
	SD	5.574	4.563	4.062	152.058
Control. group	Mean	8.7	6.3	2.3	-26.4
	Median	5	5	1	15.4
	Mode	1	1	2	50
	SD	10.893	6.748	8.103	124.997
Group comparation	Mean	1.0	-25.9	2.3	-26.4
	Median	0	0	1	15.4
	Mode	-1	0	2	50
	SD	4.062	152.058	8.103	124.997

Analysis of deviations from ideal stride length (73.5cm)							
	statistical indicators	step length		deviation from ideal step		difference	difference in %
		before therapy	after therapy	before therapy	after therapy		
Exper. group	Mean	73.3	79.0	24.7	22.1	2.6	-30.9
	Median	78	74	27.5	20.5	3	14.8
	Mode	70	50	3.5	23.5	1	46.2
	SD	29.411	26.836	15.452	15.831	16.182	143.997
Control. group	Mean	67.5	76.1	20.1	21.3	-1.2	-91.8
	Median	66	75	19.5	15.5	1	3.2
	Mode	58	72	5.5	1,5	2	0
	SD	24.019	27.638	14.189	17.598	16.241	299.718

Analysis of deviations from ideal step length between groups				
statistical indicators	Experimental group		Control group	
	difference	difference in %	difference	difference in %
Mean	2.6	-30.9	-1.2	-91.8
Median	3	14.8	1	3.2
Mode	1	46.2	2	0
SD	16.182	143.997	16.241	299.718
